# Draft genome sequence of *Rothia kristinae* AAM01 isolated from traditional yogurt in Bangladesh

**DOI:** 10.1128/mra.01388-25

**Published:** 2026-03-30

**Authors:** Md. Fakruddin, Md. Abu Bakar Karim, Samara Aliya Chowdhury, Md. Abdur Rahman, Sharmin Zaman Emon, Md. Latiful Bari, Md. Arafat Al Mamun

**Affiliations:** 1 Department of Biochemistry and Microbiology, North South University 54495, Dhaka, Bangladesh; 2 Centre for Advanced Research in Sciences, University of Dhaka 429264, Dhaka, Bangladesh; Nanchang University, Nanchang, Jiangxi, China

**Keywords:** genomes, fermented milk, Bangladesh

## Abstract

We report the draft genome sequence of *Rothia kristinae* strain AAM01 isolated from traditionally fermented cow-milk yogurt collected in Bogura district, Bangladesh, in 2023. The genome is 2,414,719 bp with a G+C content of 71.8% and assembled into 57 contigs (N50, 98,168 bp). This genome expands the available genomic resources for *Rothia* species associated with fermented dairy environments.

## ANNOUNCEMENT

The genus *Rothia* (family *Micrococcaceae*) comprises gram-positive, catalase-positive cocci commonly found in the human oral cavity and occasionally in food environments (1–3). Traditional fermented yogurts harbor diverse microbial communities that vary by region, raw materials, and processing methods. Here, we describe the draft genome sequence of *Rothia kristinae* strain AAM01 isolated from traditionally fermented cow-milk yogurt from Bogura, Bangladesh (24.85°N, 89.37°E).

The yogurt was produced by spontaneous fermentation of boiled cow milk at ambient temperature in a household setting. A 10 g sample was serially diluted and plated on M17 agar under anaerobic conditions at 37°C. Colonies were repeatedly re-streaked until pure. Acid production was confirmed by phenol red milk fermentation assay and clot formation after 24 h. The isolate was initially identified by 16S rRNA gene sequencing and confirmed by Kraken2 v2.1.3 classification and average nucleotide identity (ANI >95%) against reference *R. kristinae* genomes.

Genomic DNA was extracted using the Qiagen DNeasy Blood & Tissue Kit. Libraries were prepared using Nextera DNA Flex and sequenced on an Illumina NovaSeq 550 platform (2 × 150 bp). A total of 1,831,720 paired-end reads were generated, providing approximately 127.6× genome coverage. Read quality was assessed with FastQC v0.11.9 (4) and trimmed with Trimmomatic v0.39 (5). Genome assembly was performed using SPAdes v4.1.0 (6). Annotation was conducted using Prokka v1.14.6 (7). Taxonomic identification of the organism was performed using Kraken2 v2.1.3 (8). Functional annotation and subsystem classification were performed using RAST (9). Antibiotic resistance and virulence genes were screened using ABRicate v1.0.1 (10). Prophage regions were detected with PHASTEST (11). Roary v3.13.0 (12) was used for pangenome analysis with a 95% identity threshold using four complete *Rothia* genomes from NCBI (Accession no: SAMN39553386; SAMD00046484; SAMN51730357; SAMEA4530644). Default parameters were used unless otherwise stated.

The draft genome is 2,414,719 bp with a G+C content of 71.8%, assembled into 57 contigs (N50, 98,168 bp; N90, 23,375 bp). A total of 2,052 coding sequences were identified, including 1,272 proteins with functional assignments and 780 hypothetical proteins. The genome contains 59 RNA genes (6 rRNAs, 52 tRNAs, and 1 tmRNA).

Genes associated with lactose utilization (lacZ, lacS), lactic acid production, stress tolerance (groEL, dnaK, and cspA), and acid resistance were identified. One incomplete prophage region (~2 kb) was detected. ABRicate identified glycopeptide resistance-related genes, but no virulence genes were found.

## Data Availability

This whole genome shotgun project has been deposited at GenBank under the accession number JBSLYQ000000000. The sequenced genome is publicly available under SRA, BioProject, and BioSample numbers, which are SRR36606346, PRJNA1336685 and SAMN52081549, respectively. This genome is publicly available.
